# Melanoma Cellular Signaling Transduction Pathways Targeted by Polyphenols Action Mechanisms

**DOI:** 10.3390/antiox12020407

**Published:** 2023-02-07

**Authors:** Ecaterina Isacescu, Paul Chiroi, Oana Zanoaga, Andreea Nutu, Liviuta Budisan, Radu Pirlog, Atanas G. Atanasov, Ioana Berindan-Neagoe

**Affiliations:** 1 Research Center for Functional Genomics, Biomedicine and Translational Medicine, “Iuliu Hatieganu” University of Medicine and Pharmacy, 400337 Cluj-Napoca, Romania; 2 Ludwig Boltzmann Institute for Digital Health and Patient Safety, Medical University of Vienna, Spitalgasse 23, 1090 Vienna, Austria; 3 Institute of Genetics and Animal Biotechnology of the Polish Academy of Sciences, Jastrzebiec, 05-552 Magdalenka, Poland

**Keywords:** malignant melanoma, polyphenols, cellular pathways, signaling, cancer treatment, melanoma initiation, melanoma progression, microRNAs

## Abstract

Melanoma is the most aggressive type of skin cancer. Although different anti-melanoma treatments are available, their efficacy is still improvable, and the number of deaths continues to increase worldwide. A promising source of antitumor agents could be presented by polyphenols—natural plant-based compounds. Over the past decades, many studies have described multiple anticancer effects of polyphenols in melanoma, presenting their potential interactions with targeted molecules from different signaling pathways. However, to our knowledge, there is no comprehensive review on polyphenols-regulated mechanisms in melanoma cells available in the literature. To fulfill this gap, this article aims to summarize the current knowledge of molecular mechanisms of action regulated by polyphenols involved in melanoma initiation and progression. Here, we focus on in vitro and in vivo effects of polyphenol treatments on tumor-essential cellular pathways, such as cell proliferation, apoptosis, autophagy, inflammation, angiogenesis, and metastasis. Moreover, emerging studies regarding the well-marked role of polyphenols in the regulation of microRNAs (miRNAs), highlighting their contribution to melanoma development, are also epitomized. Finally, we hope this review will provide a firm basis for developing polyphenol-based therapeutic agents in melanoma treatment.

## 1. Introduction

The uncontrolled and invasive proliferation of melanocytes, the pigment cells from the basal layer of the human epidermis, can cause the formation of melanoma, a highly aggressive form of skin cancer with an increased metastatic capacity [1]. According to the latest statistics available, the incidence of melanoma surpassed 324,000 new annual cases, with over 57,000 deaths in 2020 only [2]. Based on clinical and morphological characteristics, melanoma can be divided into four main subtypes: cutaneous melanoma with chronic sun damage, cutaneous melanoma without the chronic sun damage, acral melanoma, and mucosal melanoma [3]. Even if melanoma accounts for less than 10% of overall skin cancers, it is responsible for up to 80% of all skin cancer-related deaths, emerging as one of the most dangerous malignancies [4,5,6].

Anticancer drug discovery has become a broad field of research in the past decades [7,8,9,10,11]. Several anti-melanoma treatments have been developed over the past decades, including surgical resection (local excision, wide local excision, lymphadenectomy, or sentinel lymph node biopsy) [12], chemotherapy, radiotherapy, targeted therapies (BRAF, MEK, HDAC, or EZH2 inhibitors), immunotherapies (immunomodulatory agents such as IFN-α, IL-2, IL-10, IL-15, and immune checkpoint inhibitors), nano-therapies (liposomal nanocarriers, nanoparticles, carbon nanotubes or dendrimers, extracellular vesicles) [13,14,15,16,17,18] and oncolytic virotherapy [19]. However, unless diagnosed in its early stages, when tumors can be surgically resected, the overall survival chances of melanoma patients drop significantly, with a life expectancy between six months to one year [20]. Though conventional treatment options (surgery and radiotherapy) have proved effective, they are limited to early localized melanoma tumors [21]. At the same time, the other strategies show a relatively poor response, mainly because of the innate chemoresistance of melanoma cells [5,17]. Therefore, more efficient and less toxic anticancer drugs for melanoma are urgently needed.

Natural compounds are a promising source of anticancer drugs, mainly due to their natural origin, lower toxicity, and increased bioavailability [18,19,20]. Polyphenols are biologically active compounds containing at least one aromatic ring with one or multiple hydroxyl functional groups attached [18,19]. Over the past decades, the interest in natural polyphenols, which refer to a large group of plant secondary metabolites widely available in plant-based foods and beverages, has grown considerably. Ranging from small molecules to highly polymerized compounds, natural polyphenols are usually divided into five classes, including flavonoids (60%), phenolic acids (30%), lignans, stilbenes, and others [20,21,22,23,24]. So far, more than 8000 natural polyphenols have been identified, and a growing body of evidence recommends their consumption for achieving various health benefits, mainly antioxidant, anti-inflammatory, and antibacterial ones [18,25]. Recently, an increasing number of studies have begun to explore these organic compounds for their positive effects in the prevention and treatment of various human disorders [26,27,28,29]. Over the past decade, many experimental results have emphasized polyphenols’ anticancer effects in different human tumors [30,31,32]. For example, in 2019, Yuan et al. conducted a research study focused on investigating the inhibitory effects and the associated underlying molecular mechanisms of resveratrol against the invasion and metastasis of colon cancer. In this regard, the authors assessed the in vitro and in vivo effects of resveratrol using AKT1 knockdown SW480 and SW620 colon cancer cells and a lung metastasis model of colon cancer (nude mice inoculated with SW480 cells). Their results showed that resveratrol and AKT1 knockdown treatment significantly inhibited colon cancer cell migration and invasion, increasing E-cadherin expression while decreasing that of N-cadherin, phospho (p)AKT1, pGSK3β, and Snail both in vitro and in vivo. These findings suggest that resveratrol may suppress the invasion and metastasis of colon cancer through a reversal of epithelial-to-mesenchymal transition (EMT) via the AKT/GSK3β/Snail signaling pathway [33]. In the same year, Wu et al. compared the activity of normal and breast cancer cell lines after an in vitro treatment with resveratrol. Transcriptome and qRT-PCR data proved that a large number of genes related to cell cycle and cellular apoptosis were differentially expressed after resveratrol administration. Their data propose 93 µM to be the IC-50 value of resveratrol that effectively inhibits the proliferation of 4T1 breast cancer cells by arresting the cell cycle and inducing cellular apoptosis [34]. In 2020, Gong and Xia found that resveratrol affects the viability and migration of A-375 melanoma cells by suppressing the AKT/mTOR pathway, thus triggering autophagy [35]. Likewise, in 2021, Silk et al. reviewed a comprehensive repertoire of studies supporting the anticancer effects of resveratrol by targeting the tumor microenvironment (TME) in prostate cancer [36]. Many natural polyphenols, such as resveratrol, curcumin, epigallocatechin gallate, etc., exhibited anticancer properties against many human tumors, including melanoma. Still, the underlying molecular mechanism is complex and may differ depending on the neoplasm type [37]. However, there is solid evidence supporting their value as anticancer compounds.

Therefore, natural polyphenols employ elevated anticancer proprieties by hijacking several critical hallmarks of cancer, such as promoting cellular apoptosis and senescence and regulating cell autophagy by inhibiting cancer cell proliferation and dissemination [38,39]. Many studies show that polyphenols modulate the activity of important key players in cancer, which lead to control of a wide variety of cellular processes subsequently described in the article, especially cell cycle arrest and responses to inflammatory processes [40]. In contrast with antitumor drugs, which are associated with various side effects that reduce the patients’ overall life quality and life span, polyphenols have attracted attention due to their multiple bioactivities and toxicity. Thereby, polyphenols can modulate specific mechanisms of action in well-investigated cancer pathways, the most targeted intracellular processes being p53-regulated mechanisms (that is a central element for cell cycle and apoptosis), MAPK pathway (central signaling cascades involved in the control of cell growth, differentiation, survival, and cell death), PI3K/Akt/mTOR pathway (which control essential cellular functions such as proliferation, transcription, translation, survival, growth survival, growth, metastasis, and drug chemoresistance) [40]. Despite the general chemical structure of polyphenols, their particularities could allow them to act differently in specific cellular pathways.For example, phenolic acids (or non-flavonoids) such as caffeic, feluric, and gallic acids are considered potent antitumor agents due to the presence of α and β unsaturation and can act as Michael acceptors. Flavones inhibit proliferation and stem-cell migration and induce apoptosis through the upregulation of p21 and p27 and the downregulation of NF-κB and PI3K/Akt pathways. Isoflavones, such as genistein and daidzein, can inhibit invasion and cell migration in a dose-dependent manner through the downregulation of FAK and the PI3K/Akt/GSK signaling pathway and modulates p21 and cyclin D1 expression. Some mechanisms exhibited by quercetin (flavonol) lead to cell growth inhibition, cell cycle arrest in phase S, induction of apoptosis through caspase-3 activation, and topoisomerase II activity in cancer cells. Stilbenoids can act through the increase in PTEN and decrease in oncogenic miRNAs [40].

Moreover, the actions of polyphenols also include the modulation of enzymatic activities in cancer and other diseases [41,42]. Concurrently, other antitumor mechanisms associated with polyphenols could imply an upturn in the cellular levels of reactive oxygen species (ROS), a decrease in the concentration of the antioxidant, interferences with various chemotherapy agents, regulation of DNA damage response (DDR) pathways, epigenetic regulation, and downsize or reverse of drug-resistant phenotype. Altogether, they represent a complex corroboration of molecular events that promote cellular stress and catabolism in cancer cells [43,44,45,46,47]. Recent findings support the role of polyphenols in the regulation of microRNAs (miRNAs), a primary class of non-coding RNAs (ncRNAs) involved in the modulation of gene expression in several diseases, including melanoma [48,49].

**Figure 1:**
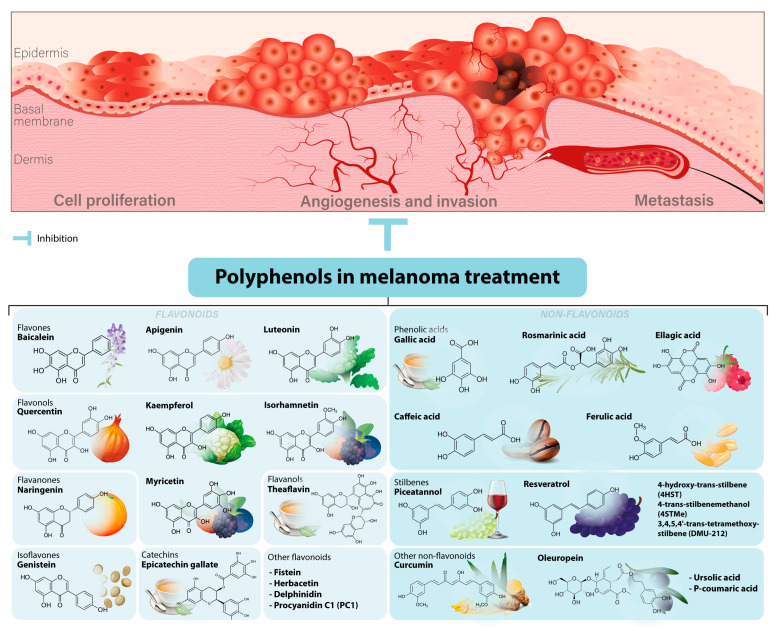
Polyphenols used in melanoma treatment. Polyphenols can inhibit the initiation and progression of melanoma through the regulation of certain cellular pathways. Chemical structures of the most common polyphenols used in melanoma treatment are given.

Over the past two decades, more than 9000 research studies have been published in several electronic databases focusing on uncovering the chemopreventive effects of polyphenols against skin cancer. Some of the most naturally abundant polyphenols include anthocyanins, ellagitannins, oleuropein dihydroxy phenyl, punicalagin, quercetin, resveratrol, and theaflavin, which were studied for their antineoplastic effects against melanoma. Several research studies have shown that polyphenols could confer photoprotection through the modulation of different cellular pathways, such as upregulation of apoptosis and autophagy and downregulation of cell proliferation, inflammation, invasion, angiogenesis, and metastasis [30,31,32,33,34]. Chemical structures of the most common polyphenols used in melanoma treatment and targeted cellular pathways are represented in Figure 1.

## 2. Effects of Polyphenols on Melanoma Initiation

### 2.1. Inhibition of Cell Growth

#### 2.1.1. Cell Cycle Arrest

Among the effect on different tumor-associated cellular pathways, the earliest observations of the anti-tumorigenic role came from their antiproliferative effects due to the inhibition of cell growth by cell cycle arrest. One of the characteristic traits of cancerous cell proliferation is the upregulation of cell cycle regulators such as cyclins and corresponding cyclin-dependent kinases (CDKs). Combined levels of their expressions favor the positive regulation of different cell cycle transitions: cyclin D-CDK4/6 complex for G1 progression, cyclin E-CDK2 for the G1-S transition, cyclin A-CDK2 for S phase progression, and cyclin A/B-CDK1 for entry into the M phase [50]. Some studies show that polyphenols can arrest the cell cycle of melanoma cells. For example, human melanoma A375 and SK-MEL-31 cells treated with resveratrol exhibited cell cycle inhibition at the G1 phase and reduction in cells in the S phase at the same time [51]. The mouse B16 melanoma cells resistant to doxorubicin showed inhibited growth in a time- and concentration-dependent manner while treated with the same polyphenol. As in the previous example, progression from G1 to the S phase was halted, supported by downregulated expression levels of cyclin D and CDKs. More specifically, the level of CDK4 was undetectable after 48 h of treatment with 50 and 100 μM of resveratrol, compared with CDK2, whose level remained unchanged after 24 h [52]. Similarly, curcumin-induced cell cycle arrest at the G1 phase in A375 cells via downregulation of cyclin D and phosphorylated retinoblastoma (RB), a tumor suppressor protein that negatively regulates the G1/S transition in the hypophosphorylated form [53]. Other studies demonstrated cell cycle arrest at the G2/M phase. In human melanoma A375 and C8161 cells, curcumin caused a reduction in G0/G1 cells with a concomitant increase in cell number in the G2/M phase [54].

Usually, polyphenols induce cell cycle arrest via upregulation of the tumor suppressor protein p53. Besides regulation of various cellular mechanisms, which are meant to prevent or stop the initiation or progression of cancer cell development, p53 mediates transcriptional activation and further upregulation of cyclin-dependent kinase inhibitor 1A (CDKN1A/p21). Similar to p21, cyclin-dependent kinase inhibitor 1B (CDKN1B/p27) is another protein found to upregulate after polyphenols treatment. Both p21 and p27 proteins play an important role in the negative regulation of cell cycle progression. Treatment of melanoma cell lines with the resveratrol analog 3,4,5,4′ -tetramethoxystilbene (DMU-212) induced upregulation of p53, its downstream effector p21, and cyclin B1 and downregulation of cyclin A2 contributing to the cell cycle arrest at the G2/M phase [55]. Likewise, A375SM cells presented G2/M blockage after resveratrol treatment. This event was associated with increased cyclin-dependent kinase inhibitors p21 and p27, contrasting with a significant decrease in cyclin B [56]. More studies have described polyphenols’ inhibitory effects on melanoma cell proliferation achieved via the regulation of cell cycle-associated proteins [42,57,58].

#### 2.1.2. Antiproliferative Effect of MAPK Pathway Regulation

Mitogen-activated protein kinase (MAPK) cascade is known to be altered in many cancer types. It is responsible for transducing extracellular signals into the cell, regulating certain cellular activities. Extracellular signal-regulated kinases (ERK1/2), p38 MAPK, and c-Jun N-terminal kinase/stress-activated protein kinase (JNK/SAPK) are the most representative MAPK kinases involved in performing different outcomes, such as cell proliferation, survival, differentiation, inflammation, or apoptosis [59]. Kim and collaborators [60] sought to determine whether treatment with quercetin on A375SM and A375P cells affects the MAPK pathway. Results showed increased p-JNK, p-p38, and p-ERK1/2 kinases associated with an antiproliferative effect. The cellular content of their unphosphorylated forms was constant during the treatment with different concentrations. Immunohistochemistry (IHC) staining of nude mice tumor tissue confirmed the upregulation of p-JNK and p-p38, although levels of p-ERK1/2 were not assessed. It was proposed that upregulation of these MAPK kinases promotes apoptosis in melanoma cells, which was supported by the expression of BAX, BCL-2, and cleaved PARP. Additionally, other polyphenols had similar effects, for example, upregulation of JNK and p-p38 was observed in A375 cells after treatment with 3,4,5,4′ trans-tetramethoxystilbene, a resveratrol analog [61].

Treatment with theaflavin induced upregulation of JNK pathway-related proteins, such as ASK1, JNK, and c-JUN, and p53 pathway-related proteins, such as ATM, CHK1/2, p53, and CASP8/3, which was further correlated with growth inhibition and intrinsic apoptosis [62]. Likewise, the upregulation of p-ERK1/2 and its translocation into the nucleus were assessed in melanoma cells that underwent apoptosis after treatment with DMU-212, another resveratrol analog [55]. Although JNK and p-p38 seem to favor apoptosis and growth inhibition, the p38 MAPK was significantly inhibited after treatment of A375, MV3, and M14 cells with curcumin [63].

Another study correlated resveratrol treatment of HT-144 melanoma cells with the inhibition of proliferation and promotion of differentiation. The antiproliferative and maturation effects of resveratrol are due to the downregulation of two enzymes from the MAPK signaling pathway: ERK and MEK 1/2. Specifically, resveratrol treatment downregulated the activity of MEK through inhibition of its phosphorylation, therefore causing changes in signaling pathways, which resulted in two important outcomes. The first is the deregulation of further MAPK signaling cascade, thus providing a secondary antiproliferative effect. The second one is the induction of melanogenesis through the upregulation of cAMP-response element binding proteins (CREB) and tyrosinase activity, both playing an important role in the process of melanin synthesis. Interestingly, microphthalmia-associated transcription factor (MITF), which is involved in the regulation of the *Tyrosinase (Tyr)* gene, and whose gene transcription is promoted by the activation of CREB, did not show a deviant expression level while treated with resveratrol. In this case, it seems that the anticancer activity of resveratrol lies in the inhibition of the MEK/ERK pathway and establishment of melanogenesis, therefore forcing melanoma cells to enter into a mature differentiated state that limits their proliferative potential [49,50]. Similar effects on the role of polyphenols-induced melanoma maturation and differentiation were conducted, highlighting their importance as a source of naturally available tumor-limiting agents [52,53,54,55,56,59,60,64,65,66].

### 2.2. Apoptosis

#### 2.2.1. p53-Dependent Apoptosis

Alongside antiproliferative effects, many studies demonstrated polyphenols’ induction of apoptosis in melanoma cells. As mentioned, their product is associated with the upregulation of tumor suppressor protein p53, which limits proliferation and induces apoptosis. At the same time, during polyphenols treatment, they can trigger either intrinsic or extrinsic apoptosis pathways. The intrinsic pathway implies regulation of apoptotic and anti-apoptotic members of the BCL-2 family, permeabilization of the outer mitochondrial membrane, and release of cytochrome *c* mitochondria in the cytosol, which induces the formation of apoptosome complex and activation of caspases 9 and 3. The extrinsic pathway assumes activation of death receptors, formation of death-inducing signaling complex (DISC), and activation of caspases, primarily caspases 8 and 3. In both paths, cascade activation of caspases leads to the cleavage of several different substrates in the cytoplasm and nucleus, resulting in apoptotic cell death with the depiction of other morphologic features [67]. Evidence of proapoptotic effects was supported by apoptotic-associated translocation of phosphatidylserine [65], formation of apoptotic bodies [68], detection of DNA fragmentation [57,63,69], and DNA double-strand breaks [63].

An early study conducted by Hsieh presented a mechanism of upstream p53 upregulation. Here, treatment with resveratrol was correlated with elevated levels of both quinone reductase 2 (NQO2) and p53, suggesting that NQO2 can potentially bind and stabilize the protein, contributing to its upregulation [70]. As in the case of A375 and SK-MEL-31 cell lines [51], it was suggested that resveratrol treatment increased the expression level of p53, which upregulated the expression of downstream proapoptotic protein BAX. Consequently, mitochondria-dependent apoptosis was induced, supported by an assessment of increased cleaved caspase 9 and 3. Concomitantly, attenuated expression of antagonistic oncoprotein BCL-2 suggested that increased expression level of the BAX/BCL-2 protein ratio plays a vital role in polyphenol-induced apoptosis.

Multiple independent studies also supported inhibitory polyphenolic effects on melanoma cells viability through upregulation of p53 and/or regulation of its downstream proapoptotic and anti-apoptotic effectors, such as BAX and BID or BCL-2, BCL-XL, and MCL-1, respectively, accompanied with elevated levels of cleaved caspases of intrinsic or extrinsic apoptotic pathways [57,62,69,71,72,73].

#### 2.2.2. p53-Independent Apoptosis

Zhao et al. [70] showed that resveratrol induced melanoma apoptosis in MV3 and A375 cell lines by negative regulation of the ERK/PKM2/BCL-2 axis rather than upregulation of p53. In cancer cells, pyruvate kinase M2 (PKM2) supports the maintenance of BCL-2 levels by inhibiting its ubiquitination. Resveratrol showed the ability to target PKM2 and its upstream activator, phosphorylated ERK1/2, by negative regulation at their mRNA and protein levels. So, a rise in BCL-2 ubiquitination resulted. Once BCL-2 is inhibited, this leads to the upregulation of BAX proapoptotic activity and induces cytochrome *c* release into the cytoplasm, following further canonical steps of the apoptotic pathway.

Some studies indicate that polyphenols do not affect the regulation of conventional oncogenic agents such as BCL-2, BCL-XL, or MCL-1, as expected. Habibie et al. [74] analyzed expression levels of six anti-apoptotic proteins, including BCL-2, BCL-XL, XIAP, survivin (BIRC5), BCL2A1, and MCL-1 in several melanoma cell lines after treatment with resveratrol. It was found that the most consistently downregulated protein in four cell lines (SK-MEL-2 cells, SK-MEL-28, UACC257, B16-BL6) was survivin, suggesting that BCL2A1, MCL-1, and BCL-XL are unlikely to be implied in resveratrol-induced apoptosis. Further investigations concluded that potential molecular mechanisms could be inhibited by resveratrol of the transcription factors that target survivin, either the β-catenin or STAT3, having lower expression levels after treatment. Interestingly, the p53 expression level was also evaluated as a transcription factor of survivin but did not show significant deviation.

#### 2.2.3. Oxidative Stress in Apoptotic Pathway Regulation

It is well known that elevated levels of reactive species in living cells can trigger tumor development and progression initiation, as they can directly damage genetic material via oxidation and invoke pathways involved in uncontrolled proliferation, growth, and survival [75]. However, the question of whether subjecting cancer cells to oxidative stress can always predispose to malignancy has risen a while ago. Results of many investigations show that oxidative stress can be one of the critical factors in promoting cellular death. While a moderate level of ROS provokes DNA mutations and triggers tumorigenesis, too-high ROS level is generally detrimental to the intracellular environment, as it activates signaling pathways of programmed cell death, providing anti-oncogenic effects [76]. Therefore, the regulation of oxidative stress became investigated as a potential mechanism of cancer therapy [77,78,79].

Polyphenols have been proposed as a valuable remedy in adjuvant therapy for many inflammatory diseases due to their well-known antioxidant properties [80]. Diverse polyphenols-based diet strategies have been encouraged to prevent cancer development by downregulating pro-inflammatory signaling pathways [81]. Nevertheless, it was suggested that polyphenols can exert double-faced actions as either antioxidants or prooxidants via the regulation of ROS-mediated pathways. While acting as prooxidants, polyphenols promote the anticancer effect and increase ROS levels, which could further regulate the pathway involving AMP-activated protein kinase (AMPK), p53, and p21 [80,81,82]. Unfortunately, it remains unclear when polyphenols turn into ROS generators or ROS scavengers. Studies indicate that the most crucial factor for this switch is the applied concentration, which can be related to differential ROS production levels. However, such factors as cell types, treatment time, metal ions, and antioxidant enzymes can also contribute to the switch [83]. Quyang et al. [84] comprehensively reviewed how EGCG provides prooxidant effects in anticancer activity and how the autooxidation process of EGCG plays a significant role. In various cancer types, it upregulated the production of ROS, such as H_2_O_2_, activated JNK, caspase-9, and caspase-3, and induced cell growth inhibition, apoptosis, autophagy, or DNA damage. Used concentration and treatment time varied from 1 to 400 µL, and from 1 to 72 h, respectively. Examination of different human cancer cell lines treated with curcumin indicated anti-tumorigenic activity, which was well-correlated with increased ROS levels over the threshold in the cell. A possible underlying mechanism lies in the binding of curcumin and curcumin derivatives to ROS metabolic enzymes, such as glutathione-S-transferase, NAD(P)H dehydrogenase quinone 1 and 2, peroxiredoxin-1, carbonyl reductase 1, and glyoxalase, therefore competing with native co-enzymes and affecting their activity [85]. Another study presented tumor suppressing activity caused by quercetin treatment in cancer cells. Polyphenol induced apoptosis through upregulation of intracellular ROS levels, leading to further activation of the sestrin 2/AMPK/p38 pathway in a p53-independent manner [86].

Studying the polyphenols treatment effects on melanoma cells, it was suggested that the resveratrol induced oxidative stress by promoting ROS formation in A375SM cells, supported by a significant decrease in antioxidant nuclear factor erythroid 2-related factor 2 (NRF2). After treatment, levels of p-p38 and p53 proteins were also elevated, suggesting ROS led to the phosphorylation of p38 MAPK and p53 activation. Consequently, the cell cycle arrest at the G2/M phase resulted via upregulation of two p53 downstream effectors –p21 and p27– and induction of mitochondria-dependent apoptosis through an increase in BAX/BCL-2 ratio. At the same time, resveratrol enhanced endoplasmic reticulum (ER) stress, confirmed by elevated levels of C/EBP homologous protein (CHOP) and phosphorylated eukaryotic initiation factor 2α (p-eIF2α), both being ER stress-associated apoptosis markers [56]. In another example, B16-F10 mouse melanoma cells treated with curcumin presented significantly increased production of reactive ROS in a concentration-dependent manner and loss of mitochondrial membrane potential. These events were associated with mitochondria-dependent apoptosis, which was also supported by an increased level of BAX/BCL-2 ratio [87]. Qiu and colleagues evaluated the role of curcumin toxicity on mitochondrial depolarization. Although levels of ROS were not assessed, authors highlighted an increase in cytosolic cytochrome C levels and opening of mitochondrial permeability transition pore (mPTP) via the association of cyclophilin-D (CyPD) and adenine nucleotide translocator-1 (ANT-1), both being protean components of the mitochondrial channel [88].

### 2.3. Autophagy

#### Regulation of AKT/PKB Pathway

A growing body of evidence indicates that polyphenols show the ability to regulate autophagy. This cell death is characterized by the degradation of cell content by the cells’ lysosomal system. Initially, nutritional starvation can induce autophagy by activating autophagy regulator kinase—unc-51-like kinase (ULK1) through phosphorylation. In its turn, ULK1 phosphorylates Beclin-1, involved in the nucleation step of autophagosome formation. Phosphatidylethanolamine moieties are added to LC3-I, contributing to the production of LC3-II, which is the primary substrate for autophagosome biogenesis. LC3-II binds the inner and outer membranes of the forming autophagosome. Cargo delivery to autophagosomes is mediated by p62, a protein that binds ubiquitinated proteins and interacts with LC3. In the final steps, autophagosomes fuse with lysosomes, resulting in cellular degradation [89]. The AKT/mTOR pathway is the most critical negative regulator of autophagy, acting primarily through the inhibition of ULK1 [90].

Wang and colleagues [91] demonstrated that treating melanoma cell line B16 with resveratrol could induce two types of cell death: apoptosis, in a caspase-dependent manner, and autophagy via ULK1 activation. Studies found that resveratrol increases ceramide production, which constitutes the initial point in the activation of the autophagy pathway. Ceramides are related sphingolipids that act as second messengers in various cellular processes, especially inducing cell death in response to different stimuli [92]. Accumulation of ceramides dephosphorylates and inhibits AKT/PKB activity, which leads to the inactivation of the mTORC1. Once mTORC1 is downregulated, the entire reaction cascade starts to accomplish autophagy. These include the activation of autophagy, activating kinases ULK1/2 and autophagy-related proteins (Atg), and raising the production of LC3-II. At the same time, a reduced level of mTORC1 favors 4EBP1 and P70S6K/S6K dephosphorylation and therefore halts protein translation. Additionally, it was demonstrated that inhibition of the autophagy pathway by treating the melanoma cells with both resveratrol and autophagic inhibitors, such as 3-methyladenine and siRNAs against Beclin 1, had increased cytotoxicity through upregulation of the apoptotic pathway. These results suggest that resveratrol-induced autophagy, accomplished through the ceramide/AKT/mTOR pathway, might function as a resistance mechanism against apoptotic cell death. Later, the downregulation of p-AKT, p-mTOR, and p-P70S6K were obtained in A375 and BRAF mutation-negative melanoma cell line C8161 after treatment with curcumin [54]. Another study [68] showed that treatment of A375 cells with *Hibiscus sabdariffa* leaf polyphenolic extract promoted autophagy through regulation of AKT/mTOR and/or PI3K class III/Beclin1 pathways. The extract induced p-AKT and p-mTOR downregulation, conversely with PI3K class III, Beclin1, LC3, P62, and ATGs such as ATG12, ATG5, and ATG9, depicting the potential contribution of polyphenolic extract in autophagy pathway triggering. The induction of apoptosis through extrinsic (Fas/FasL) and intrinsic pathways was also demonstrated. Similar results were obtained after resveratrol treatment of B16-F10 cells [35], except for significantly decreased levels of p62 and confirmed downregulated levels of PI3K.

**Figure 2:**
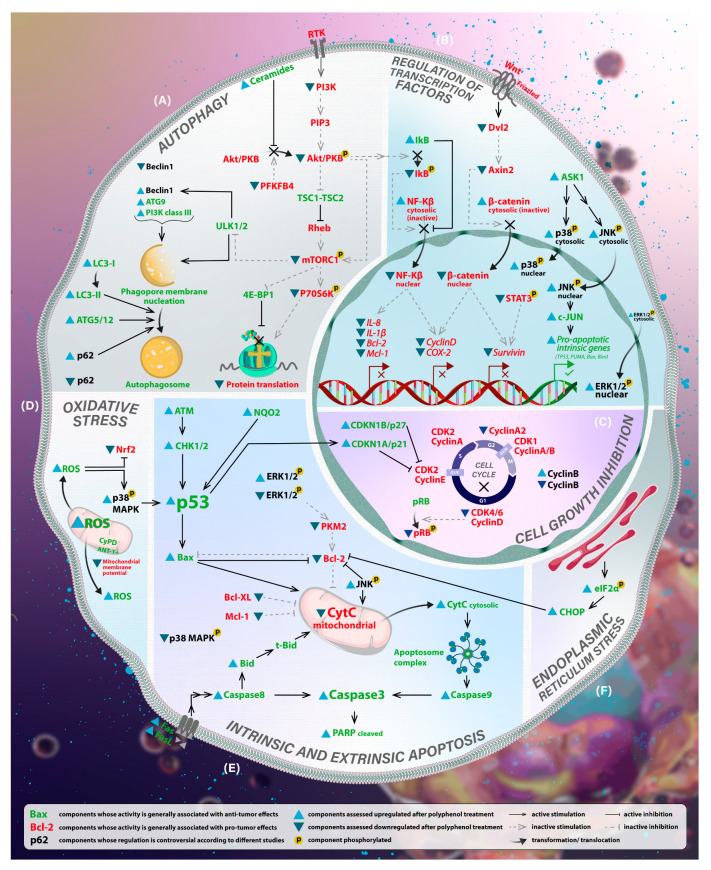
Proposed mechanisms of action of polyphenols in melanoma initiation. Polyphenols treatment of melanoma cells results in the regulation of several signaling transduction pathways. Here are depicted possible networks of cellular processes regarding melanoma initiation regulated by polyphenols, based on results from described aforementioned studies. Note that different studies demonstrated that polyphenol regulation of specific molecules, such as ERK1/2 and p38 MAPK, presented controversial results in melanoma cells. (**A**) Autophagy. PI3K/Akt/mTORC is one of the main autophagy pathways found downregulated by polyphenols. Consequently, autophagosome formation is initiated through ULK1/2, Beclin1, and ATG upregulation, which results in cellular content degradation. Additionally, mTORC downregulation leads to a halt of protein translation through the regulation of 4E-BP1 and P70S6K. (**B**) Transcription factors regulation. Polyphenols can negatively regulate the transcription of oncogenes, such as Bcl-2, Mcl-1, or Survivin, through the downregulation of certain transcription factors. For example, translocation of β-catenin in the nucleus is inhibited via downregulation of the Wnt pathway. A decrease in levels of phosphorylated Akt can contribute to transcription regulation through the inactivation of transcription factor NF-κb. Transcription of proapoptotic genes, such as TP53, PUMA, or Bax, can also be regulated by polyphenols via the upregulation of kinases, such as JNK, which in turn phosphorylates and activates transcription factors. (**C**) Cell growth inhibition. Many studies demonstrated the positive effect of polyphenols upon cell cycle arrest in melanoma cells. After polyphenols treatments, cell cycle regulators such as Cyclins are found to be downregulated, and inhibitors of cyclins, such as p21 and p27, are found to be upregulated. (**D**) Oxidative stress. Polyphenol treatment showed to increase ROS levels in cytosol. Consequently, it dysregulates more cellular processes and activates apoptosis in response to oxidative stress. (**E**) Apoptosis. Polyphenols influence the intrinsic pathway of apoptosis in many different ways, one of the most important being the upregulation of tumor suppressor protein p53. As a result, the release of cytochrome C in cytosol and activation of caspases 9 and 3 occur. A significant role is assigned to increased BCL-2/BAX ratio after polyphenol treatment, being independently confirmed by different studies. The extrinsic pathway is also influenced by polyphenols via positive regulation of extracellular signals, such as Fas/FasL, and finally via upregulation of caspases 8 and 3. A substantial increase in caspase 3 levels leads to enzymatic degradation of cellular content and cellular death. (**F**) Reticulum endoplasmic stress. Polyphenols can provoke endoplasmic reticulum stress, which negatively affects BCL-2 regulation and advantages the intrinsic pathway of apoptosis.

There are also controversial data about whether autophagy benefits anticancer therapy. Some evidence suggests that autophagy can have a protective role against chemotherapies, which in particular circumstances, can welcome the idea of autophagy inhibition in cancerous cells [93]. In this regard, Junco et al. [94] studied the treatment of B16F10 metastatic mouse and A375 human melanoma cells with polyphenols (resveratrol and ursolic acid) and their combination with chloroquine. This chemical compound prevents autophagosomes-lysosomes fusion. The study resulted in the inhibition of cell growth, apoptosis induction, and decreased autophagosome levels. Interestingly, Beclin-1 and p62 were found to reduce, in contrast to increased LC3-II levels, assuming that autophagosome formation is inhibited through beclin-1 suppression, leading to an increase in unused LC3-II. Figure 2 are presented the principal pathways regulated by polyphenols in melanoma initiation.

Representative aforementioned studies regarding the regulation of tumor initiation after polyphenols treatment are summarized in Table 1. More detailed descriptions of these and other studies [47,53,66,95,96,97,98,99,100] regarding the effects of polyphenols on cell proliferation, apoptosis, autophagy, and other cellular pathways are found in Supplementary Table S1.

## 3. Effects of Polyphenols on Tumor Progression in Melanoma

### 3.1. Regulation of Invasiveness and Metastasis

#### 3.1.1. Modulation of the Immune System

A significant role in promoting malignant cell growth, invasion, and metastasis has been assigned to inflammatory mediators [105]. Tumor necrosis factor alpha (TNF-α) is one of the inflammatory cytokines involved in inflammatory signaling pathways and is linked to malignant tumor progression [106]. Moreover, TNF-α has been reported to drive tumor eradication and progression in cancer patients, depending on the dose and cancer type [79,80]. In melanoma cells, TNFα/TNFR1 signaling mediates the significant upregulation of the pro-inflammatory cytokine IL6 gene supported by the TNF-α overexpression [107]. The adaptor protein TRADD is associated with the death domain, initiating the modulation of the MAPK and NF-κb pathways through cysteine protease caspase-8 [106]. In addition, IL-6 overexpression supports the activation of signal pathways involved in inflammation by inducing autophagy followed by premature senescence [107].

Polyphenols have been frequently reported to be involved in the inhibition of TNF-α. UV-B-mediated phosphorylation of ERKl/2, JNK1/2, and p38 protein was dose-dependent after pomegranate fruit extract treatment in normal human epidermal keratinocytes before UV-B exposure [108]. Furthermore, the treatment inhibited UV-B-mediated phosphorylation of MAPK in a time-dependent manner. Fisetin proved to be an effective inhibitor of melanoma cell invasion by promoting mesenchymal to epithelial transition and targeting MAPK, and NF-κb signaling pathways in three-dimensionally reconstituted human melanoma skin equivalents [109]. The combination of epigallocatechin-3-gallate (EGCG) and metformin has been observed to inhibit the phosphorylation levels of NF-κb p65 and STAT3 signaling pathways in melanoma cells. Thus, IL-6, IL-10, and TNF-α have been significantly decreased, inhibiting the growth and migration of melanoma cells, suggesting the potential of the two compounds tested to be used as antitumor drugs targeting inflammatory cytokines [110]. Kaempferol demonstrated significant anticancer effects on A375 human melanoma cells via induction of apoptosis, suppression of cell migration, inhibition of cell cycle arrest, and targeting of the mTOR/PI3K/AKT pathway [111]. Curcumin treatment modulated IGF-1-induced tumorigenesis in BK5.IGF-1 transgenic (Tg) mice by the phosphorylation inhibition of IGF-1 receptor, insulin receptor substrate-1, AKT, S6K, and 4EBP1 [112]. Treatment with rosmarinic acid significantly reduced cell viability, proliferation, migrative and invasive abilities, and chemotherapy sensitivity of melanoma cells through inhibition of the ADAM17/EGFR/AKT/GSK3β axis [113]. Apigenin inhibited melanoma cell migration by inducing anoikis through the modulation of integrin signaling pathways, caspase-3, focal adhesion kinase (FAK)/ERK, and cleaved PARP [114]. Genistein inhibited the proliferation and regulates melanoma cells’ migration and invasion capacity via the FAK/paxillin and MAPK pathways in a highly concentration-dependent manner [115]. Tea polyphenols inhibited the proliferation, migration, and invasion of melanoma cells in a dose and time-dependent manner through the downregulation of toll-like receptor 4 (TLR4) [116].

In contrast with pro-inflammatory cytokines, type I interferon (IFN) plays a significant role in host defense against malignant cells by triggering the immune system [117]. Taking this into account, both in vitro and in vivo studies demonstrated that quercetin inhibited cell growth, invasion, and migration in melanoma by upregulation of IFN-α and IFN-β expression. It was proposed that polyphenol acted through the activation of retinoic acid-inducible gene I protein (RIG-I) promoter, which resulted in a significant increase in RIG-I and downstream interferon regulatory factor 7 (IRF7). Increased IFN-I activated the JAK1-STAT1 signaling and contributed to IFN-I upregulation in a positive feedback loop manner [118]. Another study supported controversial results regarding the regulation of STAT1. In human and mouse melanoma cells, EGCG suppressed IFN-γ-induced JAK-STAT signaling and inhibited PD-L1/PDL2 expression. Moreover, EGCG treatment protected T-cells from collapse and increased their immune responses [119].

#### 3.1.2. Downregulation of Matrix Metalloproteinases

Other pivotal proteins responsible for tumor progression are matrix metalloproteinases (MMPs), which are zinc-binding proteases required for the degradation of extracellular matrix (ECM), contributing, therefore, to malignant cell invasiveness. Expression of MMPs is also known to be related to cellular proliferation and migration, anti-inflammatory or pro-inflammatory activity, enhancement of TGF-b and IGF-1 bioavailability, and EMT [120]. Quercetin was found to inhibit the invasion of murine melanoma B16-BL6 cells by decreasing pro-MMP-9 via the PKC pathway [121]. The downregulation of STAT3-targeted genes MCL-1, MMP-2, MMP-9, and VEGF, which promote cell growth, migration, and invasion, have been identified in melanoma cells after quercetin treatment [122]. Luteolin inhibited the proliferation and induced the apoptosis of melanoma cells by downregulating MMP-2 and MMP-9 expression through the PI3K/AKT pathway [123]. Similarly, baicalein significantly inhibited migration and invasion of melanoma cells by suppressing MMP-2 and -9 expression and activity associated with the suppression of the phosphoinositide 3-kinase/AKT signaling pathway [124].

### 3.2. Regulation of Angiogenesis

**Figure 3:**
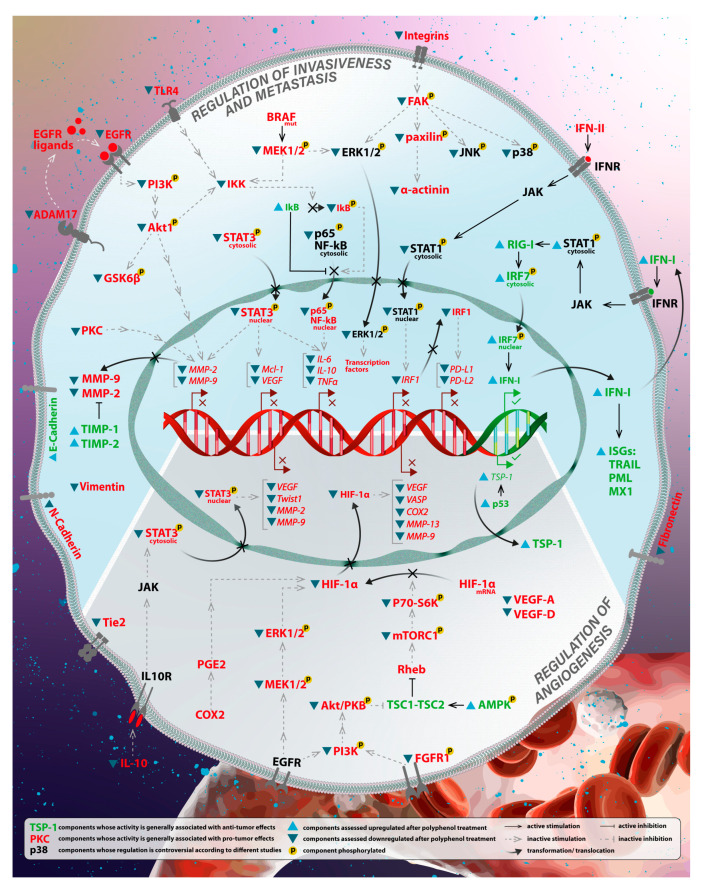
Proposed mechanisms of action of polyphenols in melanoma progression. In melanoma, polyphenols treatment of cells leads to the modulation of several signaling transduction pathways. Here are presented possible networks of cellular processes regarding melanoma progression, based on obtained results from described aforementioned studies. Note that some studies showed controversial results regarding the regulation of specific molecules, such as STAT1. (1) Regulation of invasiveness and metastasis. Polyphenols have shown to inhibit tumor progression through modulation of the immune system. For example, levels of inflammatory-related components, such as IL-6, IL-10, TNFα, PD-L1, or PD-L2, are found to decrease after polyphenols treatment via regulation of transcription factors (STAT1, STAT3, or NF-κb). Polyphenols are also involved in negative regulation of the EMT program, contributing to MMPs downregulation through PI3K/Akt or PKC pathways and suppression of vimentin, α-actinin, and fibronectin expressions, thus compromising cell motility. (2) Inhibition of angiogenesis. Polyphenols acted as positive regulators of antiangiogenic factors. One of the most essential angiogenic components is HIF-1α, which is found downregulated via several different pathways, such as PI3K/Akt or MAPK pathways. As a result, angiogenic factors (ex. VEGF-A, VEGF-D, VASP) are suppressed, contrarily with antiangiogenic factors, such as TSP-1.

After establishment, angiogenesis represents one of primordial cancer hallmarks as it supplies lacking nutrients and oxygen to a newly formed tumor, therefore allowing its survival. During numerous investigations regarding tumor progression, expression levels of pro-angiogenic regulators, such as vascular endothelial growth factor-A (VEGF-A), basic fibroblast growth factor (bFGF), and angiopoietin 1 and 2 (Ang1, Ang2), are usually increased. An important role is also assigned to MMPs, as they participate in ECM degradation and favor tumor neovascularization. Conversely, among angiogenic inhibitors can be mentioned angiostatin, thrombospondin-1 (TSP-1), as well as tissue inhibitors of metalloproteinases (TIMPs) and interferons (IFNs). Because the expression of angiogenic factors can be regulated both by differential oxygen levels and by oncogene signaling—different cellular pathways are found to be involved in angiogenesis [125,126,127]. Trapp et al. found that resveratrol has antiangiogenic effects mediated by decreased VEGF and increased TSP-1 (a downstream target of p53) expression in melanoma-endothelial cell co-culture [128]. Dietary oleuropein suppressed significant angiogenesis factors (VEGF-A) and lymphangiogenesis (VEGF-D) by decreasing the numbers of adipocytes and M2-MΦs in the B16F10 melanoma allograft model [129]. In vivo studies revealed that ferulic acid targets the FGFR1-mediated PI3K-AKT signaling pathway and causes the inhibition of melanoma growth and angiogenesis [130]. The combination treatment with resveratrol and 5-FU on B16 murine melanoma cells suppressed metastasis by inhibiting cell migration and tumor growth and angiogenesis by downregulating the expression levels of COX-2, VEGF, and VASP [131]. Myricetin treatment significantly suppressed UVB-induced neovascularization in the skin of SKH-1 hairless mice due to its capacity to inhibit HIF-1α expression by directly suppressing PI-3 kinase activity, a key target of myricetin in the inhibition of VEGF and MMPs expression [132]. Baicalein and baicalin exhibited antitumor effects on melanoma cells due to inhibiting tumor cell glucose consumption and metabolism through the mTOR-HIF-1α signaling pathway [133]. Inhibition of UVB-induced cutaneous angiogenesis after apigenin treatment was partially independent of TSP-1 in WT and TKO mice [134]. In vitro and ex vivo angiogenesis assays demonstrated that naringenin induces tumor cell death by decreasing ERK1/2 and JNK MAPK phosphorylation and inhibits angiogenesis in malignant melanoma by suppressing endothelial cell migration, tube formation, and sprouting of microvessels [135]. Caffeic acid demonstrated antiproliferative effects through the modulation of the JAK-STAT3 pathway and apoptosis in mouse skin. Furthermore, caffeic acid induced the expression of TSP-1 and offers protection against UVB-induced photo-carcinogenesis, probably through modulating the JAK-STAT3 in the mouse skin. Chronic UVB exposure decreased the expression of TSP-1 antiangiogenic protein, which is involved in inhibiting angiogenesis and proliferation [136]. In vivo study found that delphinidin treatment reduced tumor growth induced by B16-F10 melanoma cell xenograft by preventing VEGF-induced proliferation and activation of ERK 1/2 and p38 MAP kinase through a mechanism sensitive to PI3/AKT inhibitors [137]. Apigenin inhibited melanoma cell migration and invasion by downregulating STAT3 target genes (TWIST1, MMP-2, MMP-9, and VEGF). Furthermore, apigenin treatment decreased the expression of VEGF in melanoma cells suggesting the anti-metastatic effect [138]. A total of 1 mg/kg of herbacetin treatment significantly suppressed the tumor growth in A375 human melanoma xenografts. Moreover, herbacetin inhibited the angiogenesis of melanoma cells by blocking the EGFR signaling pathway, inhibiting the ERK and AKT’s phosphorylation. Matrix metalloproteinase 9 (MMP-9) was a pro-angiogenesis factor suppressed by herbacetin [139]. Figure 3 presents the principal pathways regulated by polyphenols in melanoma progression. The studies above regarding polyphenol-dependent regulation of melanoma development are summarized in Table 2.

## 4. The Relationship of Polyphenols and miRNAs in Melanoma

With over 2000 members identified in *Homo sapiens*, microRNAs (miRNAs) are a primary group of single-stranded, 18–22 nucleotide-long non-coding transcripts that play a crucial part in both transcriptional and post-transcriptional regulation of gene expression [140]. Mature miRNAs form hairpin loop assemblies, allowing them to interact with mRNAs. By partially binding to their complementary sequence at the 5′ untranslated regions (UTR), miRNAs inhibit their targets, and by binding at the 3′ UTR, they weaken mRNA stability or prevent translation [140].

In recent years, many studies have investigated and reported an interaction between polyphenol-based anticancer strategies and their effects on the expression of specific miRNAs involved in cancer development [83,140,141]. Some of these studies were conducted to identify the link between these phenol–miRNA interactions and different molecular mechanisms within the complex architecture of melanoma pathogenesis [142,143,144,145]. Thus, decoding the complex interaction between polyphenols and miRNA dynamics in cancer cells can provide us with important mechanistic insights that can be further developed in new therapeutic approaches.

Carpi et al. reported an elevated antitumor activity of oleacein, a secoiridoids abundant in extra-virgin olive oil, in human melanoma cells by influencing the expression patterns of several miRNAs related to mTOR and BCL-2 pathways. Their findings on the oleacein-treated 501Mel cell line highlight an induction of cell growth arrest by inducing apoptosis via the BCL-2 family pathway. In association with this effect, the expression levels of miR-34a-5p, miR-16-5p, and miR-193a-3p were observed. These miRNAs negatively regulate the antiapoptotic expression of BCL-2 and MCL-1. In addition, the increase in proapoptotic BAX expression and the decrease in its silencing miR-214-3p was noted. In addition, oleacein counteracts the expression of miR-34a-5p, miR-193a-3p, miR193a-5p, miR-16-5p, and miR-155-5p, which act as regulatory elements decreasing the transcriptional levels of c-KIT, KRAS, and PIK3R3, contrasting the mTOR signaling pathway, thus reducing its growth factor-like effects on 501Mel cancer cells [146]. In a similar study by Du et al., piceatannol was observed to induce the expression of miR-181a, causing a proapoptotic effect in WM266-4 and A2058 melanoma cells by suppressing BCL-2 [147].

Curcumin is a natural polyphenol derived from *Curcuma longa* L. that has proven antitumor effects on various malignancies, including melanoma [148,149]. In a research study, Zhang et al. found that a non-cytotoxic concentration of diphenyl difluoroketone (EF24), a curcumin analog, could suppress cell mobility and EMT on Lu1205 and A375 melanoma cell lines by increasing the expression of miR-33b, a direct suppressor of high-mobility group AT-hook 2 (HMGA2) [150]. Another miRNA modulated by EF24 was investigated by Yang et al. They found that EF24-mediated downregulation of miR-21 induces apoptosis of melanoma cells in vitro via upregulation of the expression of *PTEN* and *PDCD4*, which plays a part in cell cycle regulation and apoptotic pathways [151]. In a recent study, Tang et al. observed that curcumin administrated on A375 and HT144 melanoma cell lines repressed SOX10 and Notch1 while increasing miR-222-3p expression. The researchers noted that curcumin-enhanced miR-222-3p expression negatively regulated SOX-10 mRNA and ultimately inactivated the Notch pathway, thus limiting melanoma cell proliferation, migration, and invasion [152]. Finally, in a study conducted by Dahmke et al., it was observed that dietary intake of curcumin regulated the expression levels of 147 miRNAs in an engrafting mouse melanoma model. However, the most upregulated miRNA was mmu-miR-205-5p, whose expression was 135 times higher in curcumin-treated mouse melanoma cells than in controls. Thus, the overexpression of mmu-miR-205-5p modulated apoptosis and proliferation by downregulating BCL-2 and PCNA in vitro and in vivo [149].

Alsadi et al. recently investigated the effects of polyphenol-enriched blueberry preparation (PEBP) on HS 294T human malignant melanoma cells and B16F10 murine malignant melanoma cells. PEBP considerably inhibited the proliferation of skin cancer stem cells (CSCs) while reducing the formation of melanophores and upregulating miR-200s that target ZEB1 protein suppressing metastasis and migration in B16F10 murine malignant melanoma cells. Thus, the results demonstrate that PEBP possesses potent anticancer effects representing a potential chemopreventive agent for skin cancer [153].

In a research article from 2016, Yamada et al. observed that EGCG, a natural polyphenol found in green tea, upregulates miRNA let-7b expression by activating 67 kDa laminin receptor signaling led to downregulation of tumor suppression target gene HMGA2 in mouse melanoma B16, human melanoma Mewo and A375 cells. Their findings suggest that EGCG-induced upregulation of let-7b may repress tumor progression via suppression of HMGA2, thus inhibiting melanoma growth [154]. In a recent study by Wu et al., green tea polyphenols (GTPs) were shown to inhibit melanoma cell proliferation, migration, invasion, and EMT and promote apoptosis in vitro by the overexpression of a circular RNA, circ_MITF. By analyzing the underlying mechanisms, the researchers concluded that circ_MITF served as a sponge for miR-30e-3p to upregulate the level of HDAC2, which is an important regulator of gene expression, cell cycle, and DNA damage control in both normal and malignant melanocytes. As a result, GTPs suppressed melanoma progression by regulating the circ_MITF/miR-30e-3p/HDAC2 axis, revealing a potential therapeutic strategy for the human malignant melanoma intervention [155].

**Figure 4:**
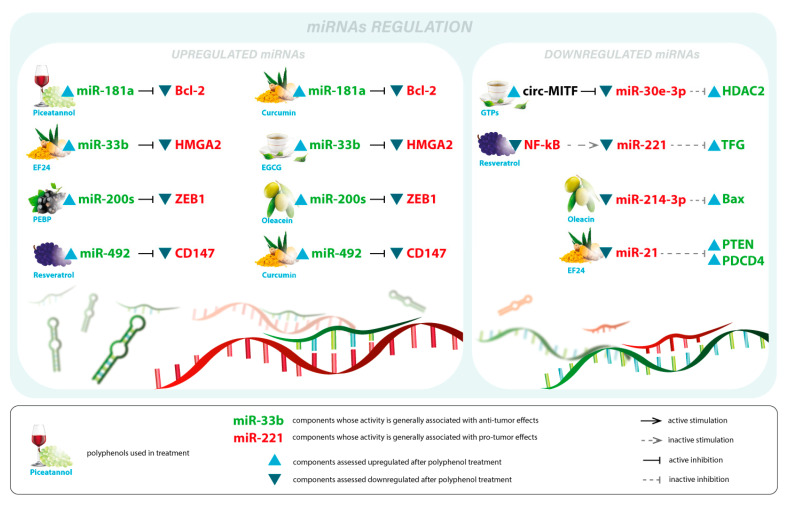
Illustrated overview of polyphenols-regulated miRNAs and their modulatory effect on key cellular events during melanoma development. This illustration summarizes the current knowledge of the regulatory mechanisms underlying the effect of natural polyphenols on miRNA expression levels, thus influencing different key cellular events involved in melanoma progression.

In an in vitro study conducted in 2017 by Wu et al. on A375 and MV3 melanoma cell lines, it was concluded that resveratrol significantly decreased the expression of the oncogenic miR-221 via downregulating NF-κb activity. Moreover, using an in vivo melanoma model, it was also noted that resveratrol-suppressed miR-221 directly induced TFG expression. Thus, these findings highlight resveratrol’s miR-221-mediated tumor suppressive activity in both in vitro and in vivo conditions [156]. In a more recent in vivo study, Zhao et al. found that post-resveratrol treatment, the inhibition rate of A375 and SK-MEL-28 malignant melanoma cell lines was significantly increased. Moreover, the upregulation of miR-492 and the inhibition of CD147 expression were also noted, these molecular changes being responsible for a strong apoptotic effect. Hence, the impact of resveratrol-induced apoptosis in melanoma cells is associated with, at least in part, its ability to regulate the miR-492/CD147 pathway [157]. Figure 4 illustrates an overview of polyphenols-regulated miRNAs in the development of melanoma.

## 5. Clinical Implications of Polyphenols and Combinatorial Therapy Approach

Nutritional interventions are becoming a standard in cancer care and prevention, with multiple studies indicating their importance and impact on prognosis and response to treatment. Multiple polyphenols widely available in vegetables and fruits have demonstrated a clear anticancer effect in melanoma, highlighting the importance of research on nutrition-based intervention as novel cancer treatment and prevention approaches [158]. In vitro and in vivo studies have proven the potential role of polyphenols as anticancer treatments in melanoma, either as individual molecules or associated with the currently available therapeutic approaches. Currently, there are no clinical trials enrolled on clinicaltrials.gov that analyze the effect of polyphenols as an anticancer drug or adjuvant in melanoma. Still, two clinical trials, NCT03950635 and NCT04645680, are investigating nutrition’s role in managing melanoma patients. These clinical trials investigate if by using nutritional interventions, we can influence the prognosis of melanoma patients or if the effect of nutrition on the microbiota can enhance the response to immunotherapy drugs. Clinical trials that focus on nutritional interventions are a promising start for the field, which indicate that soon polyphenols will pave their way to the clinic.

Resveratrol represents one of the possible polyphenols that can be the subject of dietary intervention. The anti-metastatic effect of resveratrol makes its supplementation an attractive alternative for nutritional interventions in melanoma patients [34,101]. Quercetin is a polyphenol widely available in various plants and vegetables, including onions, kale, broccoli, and tea, that, once ingested, is directly metabolized by tyrosinase in multiple compounds with anticancer activity. Quercetin was shown to be easily absorbed and metabolized, and dietary supplementation showed low or no toxicity, indicating the feasibility of using this natural compound in adjuvant or preventive strategies [159]. In addition, the administration of quercetin, as part of a combinatoric approach with green tea extract and vitamin C, showed anticancer effects in vivo xenograft cancer models [160]. Curcumin is another highly studied natural polyphenol with multiple pharmacological activities. A limitation of the usage of curcumin is represented by its low oral bioavailability, which in the last years has been surpassed by the development of novel formulations based on nanoparticles, liposomes, and micelles that increase its availability and, therefore, therapeutic effects [161]. These new formulations have the advantage of increased biocompatibility and reduced toxicity, with in vitro toxicity assays showing improved cellular viability of up to 80% in comparison with the pure curcumin extract [162].

Initial in vivo mice studies using this new curcumin formulation showed an important role for curcumin in modeling TME by reducing its tumor suppressive phenotype, inducing M1 macrophage polarization, and reducing the number of myeloid-derived suppressor cells. Additionally, curcumin showed important modulatory effects for immunotherapy, indicating a possible role as a therapeutic enhancer [161].

Another in vitro study demonstrated that a combination of ellagic acid (extracted from *Sanguisorba officinalis*) and cisplatin (a commonly used chemotherapy agent) significantly enhanced cell death in B16F10 cells [95]. Moreover, an effective decrease in AKT phosphorylation has been observed. A promising strategy to inhibit melanoma progression has been found for the combination of DM-1, a curcumin analog, and dacarbazine in B16F10 melanoma-bearing mice by the activation of apoptosis with the cleavage of caspase-3, caspase-8, and caspase-9 [163]. Co-treatment with resveratrol and 5-Fluorouracil (5-FU) reduced tumor growth in the B16 murine melanoma model by the alteration of the expression levels of AMPK, VASP, and VEGF and suppressed angiogenesis by the decrease in the number of microvascular vessels compared with the control group [131]. Combination of resveratrol treatment and TK/GCV therapy presented synergistic inhibitory effects on melanoma cells in vitro, and reduction of tumor weight and volume in vivo [97]. The combination of EGCG and vorinostat had antiproliferative effects against human melanoma cells, including modulation of the BCL-2 family proteins and NF-κb activity [164]. The combination of ECGC and metformin exhibited an antagonistic effect on cell apoptosis and oxidative stress levels in B16F10 cells [110]. The combination treatment of fisetin and sorafenib significantly inhibited the migration and invasion of BRAF-mutated melanoma cells both in vitro and in vivo and reduced the expression of EMT-inducing transcription factors [165].

## 6. Conclusions

In this study, we described the main molecular mechanisms of polyphenols involved in the initiation and development of melanoma, the most aggressive type of skin cancer. Because polyphenols are well known for their remarkable anticancer effects, particular effects of multiple polyphenol types were investigated. The most promising results were for resveratrol, curcumin, quercetin, apigenin, ellagic and rosmarinic acids polyphenols. Although the influence of polyphenol treatment on specific protein expression levels can be controversial, numerous studies indicate that polyphenols influence the expression of important bioactive proteins such as tumor suppressors, transcription factors, and cytokines. Through their regulatory role on key target proteins, they have essential anticancer activity by upregulating cellular death and downregulating cell proliferation, growth, invasion, angiogenesis, and metastasis. In addition, polyphenols have essential anti-inflammatory and anti-oxidative effects. Their nutritional supplementation has a role in cancer prevention by reducing oxidative stress and inflammation in the body, limiting the risk of de novo tumor development. Moreover, a growing amount of evidence started to emerge supporting the involvement of natural polyphenols in the regulation of specific miRNAs, thus modulating several critical pathways involved in melanoma progression. Among the most studied polyphenols, there are oleacein, piceatannol, curcumin, resveratrol, PEPB, EGCG, and GTPs, which exhibit regulatory effects on several miRNAs involved in cell growth arrest, apoptosis, EMT, proliferation, invasion, migration, metastasis, tumor growth of melanoma, mostly observed in in vitro studies. Therefore, natural polyphenols could represent a valuable and abundant source of novel therapeutic formulations against melanoma by interacting with an extensive repertoire of miRNAs and exhibiting notable antitumor effects. Providing this comprehensive review, we aimed to ensure a better understanding of molecular mechanisms and pathways that can be potentially targeted in further polyphenol-based melanoma treatments.

## Figures and Tables

**Table 1:** Effects of polyphenols on melanoma initiation.

Nr.	Compound	Cell Lines	Upregulated Targets	Downregulated Targets	Effects	Ref.
1.	Ellagic acid	1205Lu M852c A375	-	IL-1β IL-8 NF-κb	Inhibition of **proliferation** by induction of **cell cycle arrest** at G1-phase; Induction of **apoptosis**;	[101]
2.	Resveratrol	SK-MEL-2 SK-MEL-2 UACC257 B16-BL6 +In vivo	-	β-catenin STAT3 Survivin	Induction of **apoptosis**;	[74]
3.	Resveratrol	B16	Ceramides LC3-II	AKT mTORC1 4E-BP1 S6K	Induction of **mitochondria-dependent apoptosis**; Induction of **autophagy** and halt of protein translation;	[91]
4.	Curcumin	WM-115	Cytochrome C (cytosolic)	-	Decrease in mitochondrial membrane potential; Opening of mitochondrial permeability transition pore via cyclophilin-D and adenine nucleotide translocator-1 (ANT-1) association;	[88]
5.	Resveratrol	A375 SK-MEL-31	p53 BAX Caspase-9 Caspase-3	BCL-2	Reduction of cells in S phase; Inhibition of **proliferation** by induction of G1/S **cell cycle arrest**; Induction of **mitochondria-dependent apoptosis**;	[51]
6.	Curcumin	A375 MV3 M14 MRC-5	γ-H2AX t-BAX Cleaved caspase-8 (procaspase 8) Cleaved caspase-3 (procaspase 3) p53 (lower doses of curcumin)	MCL-1 BCL-2 BAX (full-length) NF-κb-p65 p38 p53 (higher doses of curcumin)	Inhibition of **proliferation**; Induction of intrinsic and extrinsic **apoptotic** pathways; Induction of DNA fragmentation, DNA double-strand breaks;	[63]
7.	Hibiscus leaf polyphenolic extract, rich in epicatechin gallate	WS1 A375 B16F10	Active-caspase-3 Active-caspase-8 Active-caspase-9 t-BID BAX Cytochrome c (cytosolic) Fas (membrane) FasL PI3K class III Beclin1 LC3-II p62 ATG5/12 conjugate ATG 16 ATG9	BCL-2 p-AKT p-mTOR	Inhibition of **cell growth**; Induction of intrinsic and extrinsic **apoptotic** pathways, also supported by apoptotic bodies formation; Induction of **autophagy**, also supported by the formation of acidic autophagolysosomal vacuoles as well;	[68]
8.	Resveratrol Ursolic acid Resveratrol + chloroquine Ursolic acid + chloroquine	B16F10 A375	LC3-II	Beclin-1 p62	Reduction of **cell growth**; Induction of **apoptosis** and decrease in autophagosomes levels;	[94]
9.	DMU-212 (3,4,5,4′-tetramethoxystilbene, a resveratrol analog)	A375 MeWo M5 Bro	p21 P53 Cyclin B1 BAX Caspase 3 Caspase 9 p-ERK1/2	Cyclin A2 p-CHK 2 BCL-2	Inhibition of **proliferation** by induction of **cell cycle arrest** at prometaphase G2/M; Induction of **apoptosis**;	[55]
10.	Curcumin	A375 C8161 +In vivo	LC3-II	p-AKT p-mTOR p-P70S6K	Inhibition of **proliferation** through cell cycle arrest in the G2/M phase; Inhibition of **invasiveness**; Induction of **autophagy**;	[54]
11.	3,4,5,4′trans-tetramethoxystilbene (resveratrol analog)	A375	JNK p-p38 p38 Aurora A	-	Inhibition of **proliferation** through cell cycle arrest in G2/M phase and prometaphase stage; Inhibition of cell **migration** and attachment to the collagen-coated surface;	[61]
12.	Resveratrol	HT-144	CREB Tyrosinase PBG-D	ERK MEK1/2	Inhibition of **proliferation**; Promoting **differentiation**;	[102]
13.	Curcumin	B16-F10 L-929	ROS BAX Caspase-3	BCL-2	Decrease in **cell viability**; Induction of **apoptosis**; Induction of DNA damage; Loss of mitochondrial membrane potential; Induction of **oxidative stress**;	[87]
14.	Resveratrol	A375SM	p21 p27 ROS p-p38 MAPK p-eIF2α CHOP p53 p-p53 BAX	Cyclin B NRF2 BCL-2	Inhibition of **proliferation** by induction of G2/M **cell cycle arrest**; Induction **ROS generation** and **ER stress**; Induction of **apoptosis**;	[56]
15.	Resveratrol	MV3 A375	Caspase3 Cleaved PARP1 p53 p53 mRNA p21 p21 mRNA BAX Cytochrome c (cytosolic)	ERK1/2 ERK1/2 mRNA PKM2 PKM2 mRNA BCL-2	Inhibition of **proliferation** in p53-independent manner; Induction of **mitochondria-dependent apoptosis**;	[70]
16.	Curcumin Quercetin	A375	Caspase 3/7 Cleaved PARP	DVL2 β-catenin Cyclin D1 COX2 AXIN2 BCL-2	Inhibition of **proliferation**; Induction of **apoptosis**;	[103]
17.	Quercetin	A375SM A375P +In vivo	BAX p-JNK p-p38 p-ERK1/2 cleaved PARP	BCL-2	Decrease in **tumor weight** (in vivo); Inhibition of **proliferation**; Induction of **apoptosis**;	[60]
18.	Resveratrol	B16-F10, A375	Cleaved caspase-9 Beclin 1 LC3II/LC3I	p62 PI3K p-AKT p-mTOR	Potential induction of **apoptosis**; Inhibition of **migration** and **invasion** and induction of **autophagy**;	[35]
19.	Isorhamnetin	B16F10 +In vivo	BAX Caspase-3 NF-κb cytosolic	BCL-2 PI3K p-AKT NF-κb nuclear PFKFB4	Inhibition of **proliferation** (in vivo and in vitro) and of **migration**; Induction of **intrinsic apoptotic pathway**;	[104]
20.	Theaflavin	A375 HFF-1 +In vivo	BAX mRNA BCL-2 mRNA Bim mRNA c-MYC mRNA p21 mRNA p53 mRNA PUMA mRNA ATM, p-ATM CHK1, p-CHK1 p-CHK2 p-P53, c-PARP ASK1, JNK, p-JNK C-JUN, p-C-JUN c-CASP8, c-CASP3	-	Inhibition of **tumor growth** (in vivo); Induction of **intrinsic apoptosis**;	[62]

**Table 2:** Effects of polyphenols on tumor progression in melanoma.

Nr.	Compound	Study Type	Model	Effects	Targets	Ref.
*Effects of polyphenols on cell proliferation, migration, and metastasis*						
1.	Pomegranate extract	In vitro	Normal human epidermal keratinocytes NHEK	Inhibition of UV-B-mediated phosphorylation of MAPK	ERKl/2, JNK1/2, and p38↓	[108]
2.	EGCG + metformin	In vitro	B16F10 cells	Inhibition of cell growth and STAT3/NF-κb pathway	STAT3 and NF-κb p65↓	[110]
3.	EGCG	In vitro	1205Lu, HS294T, and A375 cells	Inhibition of cell proliferation	STAT1↓	[119]
4.	EGCG	In vivo	C57BL/6 mice	Inhibition of tumor growth	STAT1↓	[119]
5.	Curcumin	In vivo	BK5.IGF-1 transgenic (Tg) mice	Inhibition of tumor growth	IGF-1↓	[112]
6.	Apigenin	In vitro	A2058 and A375 melanoma cells	Suppression of melanoma cells proliferation Induction of anoikis	FAK↓ ERK1/2↓	[114]
7.	Genistein	In vitro	B16F10 melanoma cells	Inhibition of cell proliferation, migration, and metastasis	p-p38, p-ERK, and p-JNK↓	[115]
8.	Quercetin	In vitro	B16 and A375 cells	Suppressed proliferation Inhibition of migration and invasion	RIG-I↑ IFN-I↑ STAT1↑	[118]
9.	Quercetin	In vivo	C57BL/6 J male mice	Inhibits tumor growth	RIG-I↑ IFN-I↑ STAT1↑	[118]
10.	Quercetin	In vitro	A375 and A2058 cells	Inhibition of proliferation of melanoma cells Suppression of migratory and invasive properties	MCL-1, MMP-2, MMP-9, and VEGF↓	[122]
11.	Fisetin	In vitro	A375 and Hs294T	Inhibition of melanoma cell invasion	MEK1/2↓ NF-κb↓	[109]
12.	Rosmarinic acid	In vitro	A375	Inhibits proliferation and migration	ADAM17/EGFR/AKT/GSK3β↓	[113]
13.	Luteolin	In vitro	A375	Inhibited the proliferation, migration, and invasion	MMP-2 and MMP-9↓ TIMP-1 and TIMP-2↑	[123]
14.	Baicalein	In vitro	B16F10 cells	Inhibits the migration and invasion of cells	MMP-2 and MMP-9↓	[124]
15.	Kaempferol	In vitro	A375 cell	Inhibition of cell migration	mTOR, phosphorylated (p) mTOR, PI3K, p-PI3K, and AKT↓	[111]
*Effects of polyphenols on angiogenesis*						
16.	Baicalein and baicalin	In vitro	Mel586 SK-MEL-2 A375 and B16F0 cells	Inhibit melanoma Cell growth and proliferation	mTOR↓	[133]
17.	Resveratrol	In vitro	Co-culture	Antiangiogenic effects	VEGF↓ TSP-1↑	[128]
18.	Oleuropein	In vivo	B16F10 melanoma allograft model	Inhibits tumor angiogenesis and lymphangiogenesis	VEGF↓	[129]
19.	Ferulic acid	In vivo	C57BL/6 mice	Growth-inhibitory activity Inhibition of angiogenesis	FGFR1↓ PI3K↓ AKT↓	[130]
20.	Resveratrol and 5-FU	In vivo	Balb/c nu/nu mice	Reduced the number of microvascular vessels	AMPK↑ VASP and VEGF↓	[131]
21.	Myricetin	In vivo	SKH-1 hairless mouse	Inhibits UVB-induced angiogenesis	VEGF↓	[132]
22.	Apigenin	In vivo	WT mice TKO mice	Inhibition of UVB-induced cutaneous angiogenesis	TSP-1↑	[134]
23.	Apigenin	In vitro	A375 and G361 cell lines, and murine melanoma B16F10 cells	Inhibition of angiogenesis	VEGF↓	[138]
24.	Naringenin	In vitro and ex vivo	B16F10 and SK-MEL-28 cells	Inhibition of angiogenesis	Tie2↓	[135]
25.	Caffeic acid	In vivo	Male Swiss albino mice	Inhibition of angiogenesis and proliferation	TSP-1↑	[136]
26.	Delphinidin	In vitro	B16-F10 melanoma cell	Antiangiogenic activity	VEGF↓	[137]
27.	Herbacetin	In vitro	A375 and Hs294T cells	Suppressed angiogenesis	MMP9↓	[139]

↓ downregulation, ↑ upregulation.

## Data Availability

Not applicable.

## Supplementary Materials

The following supporting information can be downloaded at: https://www.mdpi.com/article/10.3390/antiox12020407/s1, Table S1: Effects of polyphenols on cell proliferation, apoptosis, and autophagy.

## Abbreviations

4EBP1	Eukaryotic translation initiation factor 4E binding protein 1
ADAM17	ADAM metallopeptidase domain 17
AKT (PKB)	Protein kinase B
AMPK	AMP-activated protein kinase
ANG1/2	Angiopoietin 1/2
ANT-1	Adenine nucleotide translocator-1
ATM	Ataxia-telangiectasia mutated kinase
BAX	BCL-2-associated X
BCL-2	B-cell lymphoma 2 apoptosis regulator
BCL2A1	BCL-2-related protein A1
bFGF	Basic fibroblast growth factor
BID	BH3 interacting domain death agonist
BIRC5 (survivin)	Baculoviral IAP repeat containing 5
BRAF	BRAF proto-oncogene
CASP	Caspase
CD147 (BSG)	Basigin
CDK	Cyclin-dependent kinase
CDKN1A (p21)	Cyclin-dependent kinase inhibitor 1A
CDKN1B (p27)	Cyclin-dependent kinase inhibitor 1B
CHK1 (CHEK1)	Checkpoint kinase 1
CHK2 (CHEK2)	Checkpoint kinase 2
CHOP	C/EBP homologous protein
c-KIT (KIT)	Tyrosine-protein kinase KIT
COX-2	Cyclooxygenase 2
CREB	cAMP-response element binding protein
CSC	Cancer stem cells
CyPD	Cyclophilin-D
DDR	DNA damage response
DISC	Death-inducing signaling complex
DVL-2	Disheveled-2
ECM	Extracellular matrix
EF24	Diphenyl difluoroketone
EGCG	Epigallocatechin-3-gallate
EGFR	Epidermal growth factor receptor
eIF2α	Eukaryotic initiation factor 2 alpha
EMT	Epithelial–mesenchymal transition
ER	Endoplasmic reticulum
ERK	Extracellular signal-regulated kinase
EZH2	Enhancer of zeste 2 polycomb repressive complex 2 subunit
FAK	Focal adhesion kinase
Fas	Fas cell surface death receptor
FasL	Fas ligand
FGFR1	Fibroblast growth factor receptor
GSK3β	Glycogen synthase kinase 3 beta
GTPs	Green tea polyphenols
HDAC	Histone deacetylase
HIF-1α	Hypoxia-inducible factor 1 alpha
HMGA2	High-mobility group AT-hook 2
IFN	Interferon
IGF-1	Insulin-like growth factor 1
IHC	Immunohistochemistry
IkB	Inhibitor of nuclear factor kappa B
IL	Interleukin
IRF7	Interferon regulatory factor 7
JAK1	Janus kinase 1
JNK	JUN N-terminal kinase
KRAS	Kirsten rat sarcoma virus protein
LC3 (MAP1LC3A)	Microtubule-associated protein 1 light chain 3 alpha
MAPK	Mitogen-activated protein kinase
MCL-1	Myeloid cell leukemia 1 apoptosis regulator
MEK (MAP2K)	Mitogen-activated protein kinase kinase
miRNA (miR)	microRNA
MITF	Microphthalmia-associated transcription factor
MMP	Matrix metalloproteinases
mPTP	Mitochondrial permeability transition pore
mTORC	Mechanistic target of rapamycin kinase
ncRNA	Non-coding RNA
NF-κb	Nuclear factor kappa B
NQO2	Quinone reductase 2
NRF2	Nuclear factor erythroid 2-related factor 2
P70S6K (S6K)	Ribosomal protein S6 kinase
PARP	Poly(ADP-ribose) polymerase 1
PCNA	Proliferating cell nuclear antigen
PDCD4	Programmed cell death 4
PDL-1	Programmed death ligand 1
PDL-2	Programmed death ligand 2
PEBP	Polyphenol-enriched blueberry preparation
PFKFB4	Phosphofructo-2-kinase/fructose-2,6-bisphosphatases 4
PI3K	Phosphoinositide 3-kinase
PIK3R3	Phosphoinositide-3-kinase regulatory subunit 3
PKC	Protein kinase C
PKM2	Pyruvate kinase M2
pRB	Protein retinoblastoma
PTEN	Phosphatase and tensin homolog
RIG-I	Retinoic acid-inducible gene I protein
ROS	Reactive oxygen species
SAPK	Stress-activated protein kinase
siRNA	Small interfering RNA
SOX-10	SRY-Box transcription factor 10
STAT1	Signal transducer and activator of transcription 1
STAT3	Signal transducer and activator of transcription 3
TFG	TRK-fused gene protein
TGF	Transforming growth factor
TIMP	Tissue inhibitors of metalloproteinases
TLR4	Toll-like receptor 4
TME	Tumor microenvironment
TNF-α	Tumor necrosis factor alpha
TRADD	Tumor necrosis factor receptor-1-associated protein
TSP-1	Thrombospondin-1
TWIST1	Twist family BHLH transcription factor 1
Tyr	Tyrosinase
ULK1	Unc-51-like autophagy activating kinase 1
UTR	Untranslated region
UV-B	Ultraviolet B light
VASP	Vasodilator-stimulated phosphoprotein
VEGF	Vascular endothelial growth factor
Wnt	Wingless-Int
WT	Wild type
TKO	TSP1 knockout
XIAP	X-linked inhibitor of apoptosis protein
ZEB1	Zinc finger E-box binding homeobox 1
