# Supplementary material for: Melanoma Cellular Signaling Transduction Pathways Targeted by Polyphenols Action Mechanisms
Source: Antioxidants (Basel). 2023 Feb 7;12(2):407. doi: 10.3390/antiox12020407 (PMC9952468; doi:10.3390/antiox12020407)
Supplement: Supplementary file 1 [file antioxidants-12-00407-s001.zip › antioxidants-2125125-supplementary.pdf]

Table S1. Effects of polyphenols on cell proliferation, apoptosis and autophagy.

No.	Article title<br>Year Ref.	Melanoma cell lines	Polyphenols (treatment conditions)	Analysed molecules		General results and conclusions
				Resulted increased / upregulated	Resulted decreased / downregulated	
1.	Curcumin inhibited growth of human melanoma A375 cells via inciting oxidative stress.<br>2017<br>[53]	A375	Curcumin<br>(20, 40, 80 µM for 24, 48 and 72h)	ROS<br>Cytocrome *c* (cytosolic)<br>HIF-1α<br>BAX<br>p-AKT<br>p21<br>p53<br>p-p53<br>Active caspase 3<br>Active caspase 8<br>Active caspase 9	p-pRB<br>Cyclin D<br>MMP (mitochondria membrane potential)<br>GSH<br>G6PD<br>Cytocrome *c* (mitochondrial)<br>BCL-2	Inhibition of **proliferation** through cell cycle arrest at G1 phase;<br>Induction of **oxidative stress** by GSH depletion and mitochondria membrane potential disruption;<br>Regulation of the pentose phosphate pathway by downreg. G6PD;<br>Induction of **mitochondria-dependent apoptosis**, suggesting that ROS play an important role in it;<br>No deviant expression of:<br>E2F, Cyclin E, and CKD2;<br>LDHA, MDH1, SDH1, and IDH1;<br>BCL-XL and BID; AKT; pRB;
2.	Anti-tumor effects of resveratrol on malignant melanoma is associated with promoter demethylation of RUNX3 gene.<br>2019<br>[47]	B16F10<br>+*in vivo*	Resveratrol	p53<br>RUNX3<br>RUNX3 mRNA	hypermethylated promoter of RUNX3	Induction of **apoptosis**;<br>Inhibition of **tumour growth** (*in vivo*)
3.	Ellagic acid, extracted from *Sanguisorba officinalis*, induces G1 arrest by modulating PTEN activity in B16F10 melanoma cells.<br>2019<br>[95]	B16F10	Ellagic acid<br>Cisplatin (chemotherapy agent)	p53<br>p21<br>PTEN	p-AKT<br>p70<br>p44/42 MAPK	Inhibition of **proliferation** through cell cycle arrest in G1 phase;<br>Results suggest that ellagic acid functions as an allosteric modulator of PTEN, enhancing its protein phosphatase activity while inhibiting its lipid phosphatase activity.<br>The combination of ellagic acid and cisplatin,

					dramatically enhanced **cell death** in B16F10 cells.
4.	Procyanidin C1 inhibits melanoma cell growth by activating 67-kda laminin receptor signaling.<br>2020<br>[96]	B16	Procyanidin C1	PKA<br>PP2A<br>MRLC<br>CPI17	p-MRLC<br>p-CPI17	Inhibition of **cell growth** by activating the 67LR/PKA/PP2A/CPI17/MRLC pathway.
5.	Synergistic inhibitory effect of resveratrol and TK/GCV therapy on melanoma cells<br>2020<br>[97]	B16-TK^{+}<br>B16-TK^{-}<br>+ *In vivo*	Resveratrol<br>TK/GCV therapy<br>Combined resveratrol and TK/GCV therapy<br>(2.5, 5, 10, 20, 40, 80 µM)	Cx32<br>Cx43	-	Reduction of tumour weight and volume (*in vitro*);<br>Inhibition of **cell growth**;<br>Increased **apoptosis** of combinational treatment compared with GCV or resveratrol-alone treatment.<br>Proposed mechanism: resveratrol plays a synergistic role in increasing the inhibitory effect of TK/GCV suicide gene system through enhancement of gap junction intercellular communication (GJIC) and bystander effect of TK/GCV.
6.	Resveratrol inhibits the proliferation of melanoma cells by modulating cell cycle<br>2020<br>[66]	A375,<br>A431	Resveratrol<br>(0, 10, 50 and 100 µM for 6, 12, 24, 48 and 72 h)	-	Cyclin D1<br>PCDH9	Inhibition of **proliferation** by induction of **cell cycle arrest**, remarkably at G1 checkpoint after 24 h treatment with concentration of 50 and 100 µM.<br>Induction of **apoptosis**, being remarked that from 48 to 72 h treatment cells got through early apoptosis to death.<br>Treatment duration was established as being more vigorous factor than concentration.<br>No deviant expression of: RAC1
7.	Polyphenolic composition and anti-melanoma activity of white forsythia (*Abeliophyllum distichum* Nakai) organ extracts.<br>2020<br>[98]	SK-MEL-2<br>B16F10<br>HDFa (human dermal fibroblast)	Abeliophyllum distichum Nakai - (AL)<br>(50, 100, and 200 µg/ml for 48 h)	Casp 8<br>Casp 3<br>p-MEK1/2<br>p-ERK1/2<br>IL6R<br>CDKN1A	BCL-2<br>Casp 9<br>PDGFC<br>IGF1<br>PIK3R1<br>Akt3<br>PPP2R2B	Inhibition of **proliferation**;<br>Induction of extrinsic **apoptosis**;<br>Induction of early and late apoptosis, as well as **necrosis**;<br>Activation of the MEK-ERK pathway is involved in AL-induced **cell death**.<br>AL-induced caspase-8 and -3 might be mediated by downreg. of the PI3K/AKT pathway.<br>No deviant expression of: ROS

				NR4A1	etc.	
8.	Anti-proliferative, pro-apoptotic, anti-migrative and tumor-inhibitory effects and pleiotropic mechanism of theaflavin on B16F10 melanoma cells.<br>2021<br>[99]	B16F10<br>+*In vivo*	Theaflavin<br>(0, 40, 80, 120, 160, 200, 250, 300, 350, 400 µg/mL for 24 h and 48 h)	BAX mRNA<br>Casp3 mRNA<br>Casp8 mRNA<br>FOS mRNA<br>c-JUN mRNA<br>c-MYC mRNA<br><br>ASK1<br>p-CHK1/2<br>Cleaved Caspase 3<br>p-JNK<br>c-JUN<br>Cleaved PARP<br>p-p53	p-MEK1/2<br>p-ERK1/2<br>p-PI3K<br>p-AKT<br><br>MMP2<br>MMP9	Inhibition of **tumour growth** (*in vivo*);<br>Induction of **apoptosis;**<br>Decrease of **migrated** cells;<br>Activation of p53 and JNK signalling pathways and inhibition of MEK/ERK and PI3K/AKT signalling pathways;
9.	Antiproliferative activity of aqueous and polyphenol-rich extracts of *Larrea divaricata* Cav. on a melanoma cell line.<br>2021<br>[100]	B16F10	*Larrea divaricata* extract<br>Quercetin-3-methyl ether	-	p-STAT3	Inhibition of **proliferation**;<br>Induction of and early and late **apoptosis**;

Abbreviations

4EBP1 – Eukaryotic translation initiation factor 4E binding protein 1

ADAM17 – ADAM metallopeptidase domain 17

AKT (PKB) – Protein kinase B

ANG1/2 – Angiopoietin 1/2

ANT-1 – Adenine nucleotide translocator-1

ATM – Ataxia-telangiectasia mutated kinase

MAPK – Mitogen-activated protein kinase

MCL-1 – Myeloid cell leukemia 1 apoptosis regulator

MEK (MAP2K) – Mitogen-activated protein kinase kinase

miRNA (miR) – microRNA

MITF – Microphthalmia-associated transcription factor

MMP – Matrix metalloproteinases

BAX – BCL-2 associated X

BCL-2 – B-cell lymphoma 2 apoptosis regulator

BCL2A1 – BCL-2 related protein A1

bFGF – basic fibroblast growth factor

BID – BH3 interacting domain death agonist

BIRC5 (survivin) – Baculoviral IAP repeat containing 5

BRAF – B-Raf proto-oncogene

CASP – Caspase

CD147 (BSG) – Basigin

CDK – Cyclin dependent kinase

CDKN1A (p21) – Cyclin-dependent kinase inhibitor 1A

CDKN1B (p27) – Cyclin-dependent kinase inhibitor 1B

CHK1 (CHEK1) – Checkpoint kinase 1

CHK2 (CHEK2) – Checkpoint kinase 2

CHOP – C/EBP homologous protein

c-KIT (KIT) – Tyrosine-protein kinase KIT

COX-2 – Cyclooxygenase 2

CREB – cAMP-response element binding protein

CSC – Cancer stem cells

CyPD – Cyclophilin-D

DDR – DNA damage response

DISC – Death-inducing signalling complex

DVL-2 – Dishevelled-2

ECM – Extracellular matrix

EF24 – Diphenyl difluoroketone

EGCG – Epigallocatechin-3-gallate

EGFR – Epidermal growth factor receptor

eIF2α – Eukaryotic initiation factor 2 alpha

EMT – Epithelial–mesenchymal transition

ER – Endoplasmic reticulum

ERK – Extracellular signal-regulated kinase

mPTP – mitochondrial permeability transition pore

mTORC – Mechanistic target of rapamycin kinase

ncRNA – non-codingRNA

NF-kB – Nuclear factor-kappa B

NQO2 – Quinone reductase 2

NRF2 – Nuclear factor erythroid 2-related factor 2

P70S6K (S6K) – Ribosomal protein S6 kinase

PARP – Poly(ADP-ribose) polymerase 1

PCNA – Proliferating cell nuclear antigen

PDCD4 – Programmed cell death 4

PDL-1 – Programmed death ligand 1

PDL-2 – Programmed death ligand 2

PEBP – Polyphenol enriched blueberry preparation

PFKFB4 – 6-phosphofructo-2-kinase/fructose-2,6-bisphosphatases 4

PI3K – Phosphoinositide 3-kinase

PIK3R3 – Phosphoinositide-3-kinase regulatory subunit 3

PKC – Protein kinase C

PKM2 – Pyruvate kinase M2

pRB – Protein retinoblastoma

PTEN – Phosphatase and tensin homolog

RIG-I – Retinoic acid-inducible gene I protein

ROS – Reactive oxygen species

SAPK – Stress-activated protein kinase

siRNA – Small interfering RNA

SOX-10 – SRY-Box transcription factor 10

STAT1 – Signal transducer and activator of transcription 1

STAT3 – Signal transducer and activator of transcription 3

TFG – TRK-fused gene protein

TGF – Transforming growth factor

TIMP – Tissue inhibitors of metalloproteinases

TLR4 – Toll-like receptor 4

EZH2 – Enhancer of zeste 2 polycomb repressive complex 2 subunit

FAK (PTK) – Protein Tyrosine Kinase 2

Fas – Fas cell surface death receptor

FasL – Fas ligand

FGFR1 – Fibroblast growth factor receptor 1

GSK3β - Glycogen synthase kinase 3 beta

GTPs – Green tea polyphenols

HDAC – Histone deacetylase

HIF-1α – Hypoxia-inducible factor 1 alpha

HMGA2 – High mobility group AT-hook 2

IFN – Interferon

IGF-1 – Insulin-like growth factor 1

IHC – Immunohistochemistry

IkB – Inhibitor of nuclear factor kappa B

IL – Interleukin

IRF7 – Interferon regulatory factor 7

JAK1 – Janus kinase 1

JNK – JUN N-terminal kinase

KRAS – Kirsten Rat Sarcoma virus protein

LC3 (MAP1LC3A) – Microtubule associated protein 1 light chain 3 alpha

TNF-α – Tumour necrosis factor alpha

TRADD – Tumor necrosis factor receptor-1-associated protein

TSP-1 – Thrombospondin-1

TWIST1 – Twist family BHLH transcription factor 1

Tyr – Tyrosinase

ULK1 – Unc-51-like autophagy activating kinase 1

UTR – Untranslated region

UV-B – Ultraviolet B light

VASP – Vasodilator stimulated phosphoprotein

VEGF – Vascular endothelial growth factor

Wnt – Wingless-Int

WT – Wild-type
TKO – TSP1 knockout

XIAP – X-linked inhibitor of apoptosis protein

ZEB1 – Zinc finger E-box binding homeobox 1
